# Oxidative Stress and the Neurovascular Unit

**DOI:** 10.3390/life11080767

**Published:** 2021-07-29

**Authors:** Carmela Rinaldi, Luigi Donato, Simona Alibrandi, Concetta Scimone, Rosalia D’Angelo, Antonina Sidoti

**Affiliations:** 1 Department of Biomedical, Dental, Morphological and Functional Imaging Sciences, University of Messina, Via Consolare Valeria 1, 98125 Messina, Italy; carmela.rinaldi@unime.it (C.R.); luigi.donato@unime.it (L.D.); simona.alibrandi@unime.it (S.A.); rosalia.dangelo@unime.it (R.D.); antonella.sidoti@unime.it (A.S.); 2 Department of Biomolecular Strategies, Genetics and Avant-Garde Therapies, Istituto Euro-Mediterraneo di Scienza e Tecnologia (I.E.ME.S.T.), Via Michele Miraglia, 90139 Palermo, Italy; 3 Department of Chemical, Biological, Pharmaceutical and Environmental Sciences, University of Messina, Viale Ferdinando Stagno d’Alcontres 31, 98166 Messina, Italy

**Keywords:** neurovascular unit, oxidative stress, cell types, molecular mechanisms

## Abstract

The neurovascular unit (NVU) is a relatively recent concept that clearly describes the relationship between brain cells and their blood vessels. The components of the NVU, comprising different types of cells, are so interrelated and associated with each other that they are considered as a single functioning unit. For this reason, even slight disturbances in the NVU could severely affect brain homeostasis and health. In this review, we aim to describe the current state of knowledge concerning the role of oxidative stress on the neurovascular unit and the role of a single cell type in the NVU crosstalk.

## 1. Introduction

Although the morphological similarities between the neuronal and the vascular system have already been recognized by the Belgian anatomist Andreas Vesalius in the 15th century, only in the latest two decades the concept of neurovascular unit has emerged [1]. The neurovascular unit (NVU) represents an anatomical and functional whole which includes different cell types, which are intimately and reciprocally linked to each other and acellular elements [2,3].

Cell types include vascular cells (endothelial cells, pericytes, and vascular smooth muscle cells), glial cells (astrocytes, microglia, and oligodendroglia), and neurons. The acellular elements are proteins and enzymes that regulate the composition of the matrix. These elements are structurally and functionally integrated and interdependent and represent a highly efficient system of regulation of cerebral blood flow important to the formation and maintenance of the blood–brain barrier (BBB), which interposed between the systemic circulation and the brain parenchyma [4,5].

This interplay is made by gap junctions between components, and adhesion molecules, such as cadherins and integrins [6,7,8].

Although both neuronal and endovascular elements are critical and of vital importance, some neuroscientists considered brain cells and cerebral blood vessels distinct entities.

This led to the belief that except when the delivery of blood flow to the brain is impaired, neurons have little to do with the vascular system and vice versa.

Therefore, a rigid distinction has been made between “neurodegenerative diseases” (Alzheimer’s disease, Frontotemporal dementia, and Parkinson’s disease) and “cerebrovascular diseases” (vascular dementia, ischemic and hemorrhagic stroke). In the last decade, the knowledge about the NVU has been increased regarding structure and function. Currently, the pathophysiology and the clinical relevance of the NVU for cerebrovascular diseases and other neurologic disorders are better known. 

In fact, the NVU concept challenged previous assumptions and emphasized the symbiotic relationship between brain cells and cerebral blood vessels, taking on a central role in all aspects of normal brain function and in the pathobiology of a wide variety of brain diseases [9]. Complex communication between neural and vascular structures is required to rapidly and precisely match neuronal metabolism to blood flow within the central nervous system. The neurovascular coupling is a mechanism by which active neurons signal blood vessels to change their diameter and has become increasingly used as a method to evaluate the health and function of the cerebrovascular system and responsiveness of the neurovascular unit. 

A number of important studies have identified astrocytes as key intermediaries in neurovascular coupling, following their skill to modulate vascular tone. Astrocytes, via a Ca^2+^-dependent mechanism, engage numerous signaling pathways which lead to the release of vasoactive signals capable of both dilating and constricting arterioles. The kinetics and effectiveness of these signals are, in turn, determined by the metabolic state of the tissue, the level of basal arteriole tone, and/or the magnitude and type of stimulus evoked [10]. Deficits in components of the neurovascular unit have been linked to a wide range of conditions associated with a deteriorating central nervous system structure and function. Neurovascular coupling prepares to become a biomarker in the early detection and monitoring of most neurovascular complications observed in humans [11].

The NVU is prone to protective and damaging events, leading from health to illness, recovery, or death. In this context, systemic, endocrine, neural factors, and oxidative stress can transform all or part of the NVU. Disorders of the NVU can contribute to the initial stroke pathology and the development of a brain injury after a stroke. 

Chronic injury to the small penetrating arteries is the leading cause of a lacunar stroke, oxidative stress, vascular dementia, and intracerebral hemorrhages. 

Low microvascular density or microvascular dysregulation is likely a major factor that will contribute to eventual brain injury after a large vessel stroke. In the context of an acute stroke due to embolism or thrombosis of the large arteries, the dysfunction of the NVU is also partly responsible for cerebral edema and may lead to the progression of the lesion into the ischemic penumbra [12,13,14]. 

Therefore, understanding the mechanisms of neurovascular dysfunction in disease conditions may allow the development of potent and effective therapies for the prevention and treatment of brain diseases.

## 2. NVU and Its Major Components 

The NVU is composed of cells from the brain endothelium that interact with pericytes, astrocytes, oligodendroglial cells, microglial cells, neurons, and extracellular matrices in order to form the blood–brain barrier.

### 2.1. Pericytes

Among the NVU components, mural cells as the pericytes which are in close proximity to endothelial cells, play a vital role in the BBB integrity [15] and regulate the BBB formation and maintenance through secreting inhibitory signals. They control permeability gradients and segregate blood from the brain parenchyma [15,16,17,18]. It is yet unknown how pericytes regulate the vesicular pathways across the BBB [19] since the number of vesicles as caveolae and the rate of endothelial transcytosis are lowered. Ben-Zvi and colleagues have identified MFSD2A as a negative regulator of transcytosis in the BBB endothelial cells. Increased expression of this protein turns out to coincide with the onset of endothelial barrier function in mouse embryos and this expression was higher in the BBB endothelial cells compared with peripheral vessels. Under pathological conditions [20,21,22], pericytes are highly susceptible to oxidative stress and apoptosis [23,24,25,26].

Reactive oxygen species (ROS) are important modulators of cellular functions in pericytes. Although there are many enzymes that can potentially produce ROS, the NADPH oxidase (Nox) family proteins are the predominant source of reactive oxygen species under physiological and pathological conditions. Nox4 is a major superoxide-producing enzyme among the Nox family in human brain pericytes. Nox4 expression level was significantly increased under hypoxic conditions both in vivo and in vitro. Upregulated Nox4 increases the production of MMP-9 probably through NFkB in pericytes and plays a harmful role in the acute phase of ischemic stroke by increasing the size of the infarct area and enhancing the breakdown of the BBB. [27,28,29].

### 2.2. Endothelial Cells

The BBB endothelial cells are presented with a highly sophisticated junctional complex consisting of adherens junctions (AJs), gap junctions (GJs), and tight junctions (TJs) and exhibit an extremely high level of continuity, thereby stringently limiting paracellular diffusion of solutes and water between the blood and the brain [30,31,32,33,34,35,36]. In fact, an important feature of the BBB endothelial cells is having low rates of nonspecific transcellular vesicular transport (transcytosis), including (macro) pinocytosis, clathrin-dependent and caveolin-dependent endocytosis. Although endothelial cells have a paucity of plasma membranes invaginations, clathrin-coated vesicles present in the capillary endothelium mediate mainly transcytosis in the direction of blood-to-brain [37,38,39,40]. In this way, the BBB regulates the entrance of important substances from the bloodstream, such as glucose, and blocks the entry of neurotoxins, such as fibrinogen and peripheral immune cells, maintaining the specialized composition of the brain interstitial fluid [41,42]. In contrast, caveolae are considered negligible in the BBB integrity and seem to be the major transcellular pathway of loss of the BBB function that turns out to increase its permeability in pathological conditions [43,44]. In the last decade, several studies have raised considerable interest for the extracellular vesicles, named exosomes, which can mediate cell-to-cell communication in a variety of biological processes [45,46], as in CNS inflammation, neurogenesis, or neuroprotection [47] and as an efficient transport system of exogenous siRNA across the BBB [48,49,50,51].

In the BBB endothelial cells, Ca^2+^ signaling has an important role in enhancing the BBB permeability, and gap junctions (GJs) are also intimately involved in the Ca^2+^ signaling processes [52,53,54]. The GJs directly connect the cytoplasm of adjacent endothelial cells and facilitate the transfer of ions, metabolites, and second messengers. To date, most of the evidence available points to Ca^2+^ as an important regulator of the paracellular route, contributing to increased vesicular trafficking across the BBB. Indeed, in the BBB endothelium, the junctional protein, as well as the cytoskeletal components, appear to be important targets of the effector protein that are activated downstream of the Ca^2+^ signaling processes [54]. In fact, in the BBB endothelial cells, different connexin (Cx) and pannexin (Panx) proteins form large conductance channels in the endothelial membrane, which can facilitate direct transcellular transport. An endothelial Ca^2+^ increase is a common feature of various pathological conditions that are associated with BBB dysfunction [35,36,54,55,56,57,58,59]. Their opening is promoted by several types of cellular stress, including mechanical stress during bone remodeling [60], ischemia [61,62], subarachnoid hemorrhage [63,64], and oxidative stress [65]. 

Novel investigations have recently reported whole gene expression profiling of brain EC, aiming to search for relevant mechanisms regarding the BBB and NVU pathophysiology and for potential treatment options [66].

The endothelial cells of brain vessels (including capillaries) integrate and respond to myriad intravascular, vascular, and extravascular signals. These cells are viewed as a key regulator of blood flow, transport, BBB function, and immune surveillance of normal brain tissue. The endothelial cells have ongoing direct interaction with extravascular (astrocytes, microglia, neurons), vascular (pericytes, smooth muscle), and intravascular (leukocytes, platelets, red blood) cells [12]. It is known that the BBB is a tight barrier for water-soluble molecules that may only enter the CNS via specific transporters. Thanks to the presence of tight junctions among endothelial cells, to the absence of macropinocytosis, and to the loss of fenestrae, the BBB is a highly selective barrier. In pathological conditions, especially in the oxidative stress, the modification of these three elements alter the BBB integrity, preventing the production of an ultrafiltrate through the brain’s capillary bed. 

In particular, free radicals can directly compromise the BBB integrity via modulation of tight-junction/cytoskeletal integrity [67,68,69], disrupting both microtubules and actin filaments via matrix metalloproteinase activation [70] and via cellular communication [71]. In neurons, microtubules are of primary importance since they maintain these cells polarization and intracellular trafficking. Particularly, a cytoskeleton-associated protein, MICAL (molecule interacting with CasL) binds actin filaments and selectively oxidizes Met 44 and Met 47 in a NADPH-dependent, in order to cause filament severing [72] with consequent cell morphology alteration, to regulate blood vessel sprouting [73]. In addition, collapsin response mediator protein 2 (CRMP2) is sensitive to oxidation in cells exposed to hydrogen peroxide (H_2_O_2_) [74]. 

CRMP2 is an important regulator of microtubule stability, and through oxidation and phosphorylation it promotes its dissociation from tubulin and microtubule collapse. 

Several studies suggest that oxidative stress and consequential endothelial dysfunction have a critical role in age-related cerebromicrovascular impairment and neurovascular uncoupling [75,76,77,78,79] in different diseases [80,81,82,83]. 

For this reason, along with inflammation, oxidative stress seems to be one of the main inducers of neurodegeneration, causing excitotoxicity, neuronal loss, axonal damage, and promotes cell death. The role of neuroinflammation in many diseases, such as cerebral cavernous malformation (CCM), has been already confirmed [84], but Scimone et al. have suggest novel mechanisms involved in CCM development, through expression profile studies in CCM endothelial cells without functional deficits at the three CCM genes [85]. Studies in vivo and in vitro on mice have also used lipopolysaccharide (LPS) to induce inflammation and the destruction of the BBB. It has been seen that the BBB is relatively resistant to the LPS-induced disruption and appears to be dependent on cyclooxygenase (COX) but not on oxidative stress.

Some brain regions result to be more vulnerable than others; in fact, it appears that astrocytes and pericytes play a minor role in BBB disruption LPS-mediated [35,86,87]. Inflammation also can induce BBB disruption by altering tight junction function, because occludin and claudin-5 appear susceptible to redox changes [88,89]. Both occludin and claudin-5 contain highly conserved redox-sensitive cysteine residues that form disulfide bonds critical to the structure and function of the TJs, thus promoting paracellular opening [90,91] and transcytotic leakage [92,93].

### 2.3. Astrocytes

Astrocytes, the most abundant type of neuroglia cells are also responsible for regulating transcellular transport across the BBB. Distinct types, including radial astrocytes, fibrous astrocytes, and protoplasmic astrocytes are within the CNS based on structure, distribution, and function, as well as their expression level of the different isoforms and splice variants of the intermediate filament protein, glial fibrillary acidic protein (GFAP) [94,95]. Astrocytes have been implicated in maintaining water and ionic homeostasis, favoring the generation of action potentials in neurons protecting them against oxidative stress associated with their high energy consumption [96,97]. Astrocytes undergo a senescence-like stress response, named “astrosenescence” by Cohen and described as a functional change that has a severe impact on neurodegenerative diseases [98]. Astrocytes play a role in regulating the BBB redox homeostasis, through antioxidants release as glutathione (GSH) [99] and nuclear factor erythroid 2-related (Nrf2)-dependent glutathione [100,101]. Nrf2 is an important example of mitochondrial ROS signaling, which leads to nuclear gene expression changes [102]; in fact, Nrf2 is transferred from the cytoplasm to the nucleus, where it binds the DNA antioxidant response element (ARE) of the genes involved in the antioxidant response [103] and induces secondary defense proteins in oxidative stress condition. This allows astrocytes to be more protected than neurons against moderate levels of oxidative stress [56,57,58,104,105,106]. Astrocyte are provided with numerous protrusions that anchor neurons to their blood supply forming a complete layer around cerebral blood vessels known as glial limitans. They regulate immune cell entry, waste clearance, and blood flow [107]. Reactive astrocytes carry mitochondria to their end-feet, driving vascular remodeling, potentially through ROS generation and redox signaling [108]. 

Astrocyte end-feet also release growth factors involved in the formation of the TJ protein, such as the vascular endothelial growth factor (VEGF), which is likely a key protein in this process through its ability to affect both vascular and neuronal cells [61,62,63,64,109,110,111,112]. ROS are also able to enhance directly the VEGF transcriptional level in the brain endothelial cells exposed to oxidative stress or thrombin [113]. In fact, once bound to its receptor VEGFR2, VEGF promotes the proangiogenic signaling cascade, leading to the activation of the ERK/MAPK pathway [114,115]. 

A highly important signaling molecule, by which astrocytes both communicate with each other and with vascular cells, is also ATP and its metabolites, adenosine, and ADP [116,117]. When ATP is released from astrocytes in response to neuronal activation, it contributes to microvascular dilation by triggering the production of endothelial NO [116]. ATP is directly linked to astrocyte metabolism, but in aging, cellular energy metabolism, and ATP production are altered in different types of cells, although little is known about age-related alterations in neurovascular coupling mechanisms and astrocyte dysfunction. In any case, neurovascular uncoupling is reversible in aging by interventions that improve endothelial function and cerebromicrovascular reactivity. 

### 2.4. Oligodendrocytes

Oligodendrocytes are one of the major cell types in the white matter, and within the CNS, they produce a lipid-rich membrane called myelin to enwrap axons for efficient conduction of electrical impulses. This myelinating action is most essential during the nervous system development, but it is also critical in the repair of damaged white matter in the adult brain. It is well known that oligodendrocytes can signal to neurons via myelin–axon interactions.

Recently, it has been demonstrated that oligodendrocytes metabolically support neuronal axons with a particular reference to lactate supply. Furthermore, endothelial–oligodendrocyte interactions may contribute to ongoing angiogenesis and oligodendrogenesis in adult white matter, particularly after brain injury. During the chronic phase after a white matter injury, matrix metalloproteinase (MMP)-9 from oligodendrocytes may promote vascular remodeling [118].

### 2.5. NVU in White Matter

Although, the NVU concept has been mostly utilized referring to functional cellular units in “gray matter”, cell–cell interactions could be also very important for white matter. 

In fact, many review articles focus on astrocytes which are interposed between neurons and microvessels. In white matter, however, neuronal axons are covered by myelin. Since oligodendrocytes do not contact microvessels directly, the NVU in white matter should be considered as “neurons–oligodendrocyte–astrocyte–microvessels”. 

White matter is particularly vulnerable to cerebrovascular injury. This vulnerability has been attributed, in part, to the nature of cerebral vascular anatomy. However, the active and contributing role of glial cells in white matter vascular injury has been demonstrated by the inhibition and modulation of these cells in recent experimental models [119].

### 2.6. Microglia

In the early stages of development, microglia arise from the yolk sac and settle in the brain as the first glial cells, developing in conjunction with neurons in highly plastic cells with mobility. Under physiological or pathological conditions, the microglia continuously monitor the surrounding environment and always responds in the first place to any insult in the SNC.

The intricate relationship existing between the different components of the NVU is expressed very well with regard to microglia, capable of polarizing into distinct pro-inflammatory (M1) or anti-inflammatory (M2) phenotypes. Current data indicates that M1 pro-inflammatory microglia contribute to a BBB dysfunction and a vascular “leak”, while M2 anti-inflammatory microglia play a protective role at the BBB [120].

Over the past few decades, microglial cells have been regarded as the main executor of inflammation after acute and chronic central nervous system (CNS) disorders, responding rapidly to exogenous stimuli during acute trauma or infections, or signals released by cells undergoing cell death during conditions such as a stroke, Alzheimer’s disease (AD) and Parkinson’s disease (PD) [121,122].

### 2.7. Basement Membrane

The basement membrane (BM) represents the extracellular matrix (ECM) found predominantly underneath endothelial and epithelial cells. The ECM is a structure in constant morpho-functional “remodeling”, both in physiological and pathological conditions, based on the functional requests coming from its own interior (through the action of metalloproteases) and from the cells (through the action of adhesion proteins) [123,124]. In the brain, two types of BM are found: an endothelial BM and a parenchymal BM, separated by pericytes. Under physiological conditions, the two BM layers are indistinguishable. The main components of the BM are: collagen IV, laminin, entactin, and heparan sulfate proteoglycan 2, which have a crucial role in vascular integrity. The BM exerts many important functions, including structural support, cell anchoring, and signaling transduction [125]. Metalloproteases (MMPs) are a family of endopeptidases containing zinc and calcium, that show the potential to degrade the protein of the ECM. In particular, the 9 isoform (MMP9) is important in the brain’s microvascular environment. 

Metalloproteases activated by ROS degrade the BM protein, suggesting that oxidative stress increases the BBB permeability and promotes extravasation of inflammatory factors and ROS in the brain, causing neurodegeneration diseases [126,127,128,129]. 

## 3. Oxidative Stress and Transporters at the Neurovascular Unit

The NVU transporters are vital for the regulation of normal brain physiology. In pathological conditions, brain edema increases due to the alteration of ion, water, and glutamate transporters, primarily in astrocytes. Drug toxicity is enhanced because of the alteration of efflux transporters (ATP-binding cassette (ABC) transporters). In addition, the energy metabolism results altered because the glucose transporters are dysregulated. 

### 3.1. ABC Transporters

The brain is lipid-rich compared to other organs, but there is very little known about the potential roles for ABC transporters in brain lipid transport. ABC transporters are localized on the blood-facing plasma membrane where they allow unidirectional transport from the cytoplasm to the extracellular space. To date, 48 ABC transporters are known in the human genome. Their principal role is to extrude metabolic waste into the blood and form a selective barrier in order to protect the CNS by limiting entry of xenobiotics, including toxins and a large number of drugs [130,131]. Several genetic diseases are caused by ABC transporter mutations. For example, mutations in ABCA4, whose protein transports retinylidene phospholipid complexes within the rod outer segment of the retina, can cause Stargardt disease and other related eye disorders [132,133]. Others ABC transporters are expressed in the human brain [134,135] with specialized functions in terms of their location at the level of brain region (e.g., ABCA7 in hippocampus), cell type (e.g., ABCA2 in oligodendrocytes), and organelle (e.g., ABCD1 in peroxisomes). ABC transporters have evolved to counteract oxidative stress; indeed, ROS may function as an endothelial signal transduction intermediate promoting cell survival, increasing ABC expression to compensate the increased load of oxidative stress products, or to compensate for the loss of efflux pumps in damaged tissues [136]. Oxidative stress may also lead to increased lipid peroxidation, which is implicated in BBB disintegration, with consequent decrease in ABC expression and activity.

### 3.2. Glucose Uptake 

The brain has almost no storage of glucose, and it must be supplied continuously via the blood circulation [137,138]. Glucose uptake occurs in neurons and astrocytes via different glucose transporters (GLUTs), that are, respectively, GLUT3 and GLUT1 [139,140]. Through GLUT1, glucose supplied by the cerebral circulation can cross the BBB. 

Astrocytes play a pivotal role in glucose metabolism. Acute and chronic high-glucose environments activate the glutathione/pentose phosphate pathway (PPP) system in astrocytes, preventing ROS elevation. Chronic high-glucose environments induce endoplasmic reticulum stress through increased hexosamine biosynthetic pathway flux [141].

Astrocytes contain small amounts of glucose in the form of glycogen granules [142]. 

Unfortunately, glucose derived from astrocytes glycogen cannot cross the neural cell membrane because of its low lipid solubility and, therefore, it cannot be available for neurons. 

On the other hand, lactate or pyruvate, the end-products of glycolysis, can exit from astroglia via monocarboxylate transporter 1 (MCT1) and MCT4 and can re-enter into neurons via monocarboxylate transporter 2 (MCT2) and they can be used as an energy source for neuronal tricarboxylic acid (TCA) cycle substrates [143,144,145,146].

The metabolic roles of lactate in brain function have long been debated in the literature as an astrocyte–neuron lactate shuttle. Various models attempted to quantify the magnitude of lactate trafficking from astrocytes to neurons, from neurons to astrocytes, and between astrocytes via gap junctional transfer and lactate release from the brain.

Strong evidence points for a glutamate-evoked glycolysis in astrocytes coupled with lactate shuttling in neurons.

The cellular sources of lactate in the brain remain to be established, and neurons have many specific functions fulfilled by glycolysis, so it is risky to assume that lactate is always astrocyte derived. Recent discoveries that revealed novel signaling functions for lactate are significant advances [147,148].

## 4. Oxidative Stress and Mitochondrial Dysfunction

It is known that proper neuronal activity entails high amounts of energy. In the brain, the amount of energy is very reduced, whereby constant supply of energy substrates are required through blood flow [149]. The NVU maintains energy substrates essential to the fulfilment of the metabolic needs [150], not only through the communication among cell types but also through the mitochondria [151,152]. Mitochondria are fundamental in cell homeostasis due to their involvement in several vital processes, such as cell growth and differentiation, cell cycle control and death [153,154], intermediary metabolism, Ca^2+^ homeostasis and signaling, and apoptosis [155]. Oxidative stress can affect the mitochondrial respiratory chain function, thereby altering the membrane permeability and calcium homeostasis, along with increasing the heteroplasmic mtDNA and weakening the mitochondrial defense systems [156,157]. Complexes I and III produce radicals, as superoxide anions and hydrogen peroxides, which have various cellular signaling roles and are necessary for cell differentiation, proliferation, survival, and adaptive immunity responses. In the complex IV and during oxidative phosphorylation reactions, the movement of electrons results in the reduction of oxygen to water. Almost all the generated superoxide anions are effectively neutralized by superoxide dismutase (SOD) in order to form hydrogen peroxide, which serves as an important precursor for other free radicals and acts as a secondary messenger with the ability to diffuse across the mitochondrial membrane, through a specialized protein from the aquaporin family. In this way, ROS can modulate the expression of several genes involved in the signal transduction [158,159,160,161,162,163,164,165,166,167]. The effects related to ROS signals are different and even opposite, depending on their concentration. At elevated concentrations, they influence the oxidative stress, ultimately leading to cell death, whereas at lower concentrations, they mediate redox signaling events in favor of the progression of disease [168,169] and promoting proliferation, invasiveness, angiogenesis, and metastasis [170,171,172]. However, despite the presence of mitochondria, the endothelial cells obtain a large proportion of their energy from anaerobic glycolytic metabolism [173]. This suggests that mitochondria serve primarily as essential signaling organelles rather than as manufacturers of ATP in the vascular endothelium [174,175,176], leading the liberation of vasoactive factors from the endothelium for modulation and maintenance of the BBB integrity and brain homeostasis [177]. 

## 5. Possible Therapeutic Target to Protect the NVU

In the last years, preclinical and clinical approaches were designed to protect the NVU. Several molecules with potential therapeutic effects were identified to target ROS, boost mitochondrial function, and decrease free radical production and oxidative damage [178,179], in an attempt to improve neurovascular health. 

Noncoding RNAs are implicated in a wide variety of cellular processes, as well as in many disease conditions [180,181,182,183,184,185,186,187] in which the oxidative stress alters ROS homeostasis. Oxidative stress induces the expression of several transcription factors such as NFκB, AP-1, p53, HIF-1α, PPAR-γ, β-catenin/Wnt, and Nrf2 that, in turn, activate the expression of hundreds of genes which are associated with growth factors signaling, immune responses, and cell cycle transition. Some lncRNAs that are aberrantly expressed in oxidative stress have functional interactions with miRNAs especially in the neurodegenerative conditions [188]. Therefore, these transcripts could be regarded as biomarkers for the assessment of the levels of oxidative/antioxidative imbalance [189].

In case of intracerebral hemorrhage in combination with intraventricular hemorrhage (IVH), intraventricular recombinant tissue plasminogen activator (rt-PA) represents a good approach to dissolve the blood clot in the ventricular system. However, blood derivatives enter the parenchyma and may still adversely affect functional structures of the brain [42]. In contrast, clinical arterial approaches to lowering blood pressure (and, therefore, cerebral reperfusion pressure) in an acute ischemic stroke have not shown clear benefits [190]. One clinical study demonstrated that lithium exposure in bipolar disorder determines a reduced risk of stroke, potentially being able to protect the endothelium and NVU [58]. Experimental studies suggested that an endothelial Ca^2+^ overload plays a role in endothelial dysfunction including the BBB opening, a key mechanism in the acute stage of a stroke; therefore, lithium significantly increasing the stability of the BBB [35,36,57]. Through the inhibition of the activity of the phosphoinositol phosphatases, it decreases levels of inositol 1,4,5-trisphosphate, by resulting in possible induction of autophagy [191].

Also the Xestospongin C, a potent inhibitor of the Ins(3)P-sensitive release channel that displays high selectivity over ryanodine receptors, suppress the early Ca^2+^ rise in metabolically inhibited endothelial cells [192].

Low-dose therapeutic lithium concentrations significantly augment the cerebral vessel relaxation, independently of central and autonomic nerve system influences and also stabilized the dynamic thrombin-induced and PAR-1 receptor agonist-induced permeability of human endothelium [35]. Instead, lithium accumulation or overdose reduces endothelium-dependent but not endothelium-independent vasorelaxation, suggesting that lithium could have differential effects on the endothelium and the vasculature [193].

Recent clinical trials have demonstrated the efficacy of glibenclamide, a drug that binds the sulfonylurea receptor 1 proteins at potassium channels and may significantly reduce cerebral edema following a stroke [194].

Intravenous glyburide that reduces brain swelling and improves survival in preclinical models of a stroke has shown to be effective also in patients who suffered an ischaemic stroke and a large hemispheric infarction [195].

## 6. Conclusions

The brain is one of the main organs in the body with the highest metabolic demand and requires a tight regulation of the surrounding environment.

This tight control is exerted by the NVU, comprising different cell types. Even slight perturbations in the NVU might affect, in some cases irreversibly, brain homeostasis and health.

It is also known that in many cases the BBB integrity is deeply affected by oxidative stress. In fact, increased reactive oxygen species (ROS) production contribute to endothelium dysfunction and increased permeability of the BBB [196].

Together with oxidative stress, several pathological factors can cause BBB compromise, mainly increasing BBB permeability. 

Direct damage to endothelial cells and the BBB can affect other components of the neurovascular unit, further aggravating BBB damage and dysfunction and eventually leading to neuronal dysfunction, neuroinflammation, and neurodegeneration. 

Studying the role of oxidative stress in the pathogenesis of neurological diseases and protecting the BBB in the early stages can be a great help in limiting the progression of the disease.

About the antioxidant’s effects of a specific target, to date, the evidence is limited in human endothelial cells [35,87,197], and the use of antioxidant therapy in cerebrovascular disease still needs further details.

## Data Availability

The study did not report any data.
